# Death Certificates and Murder

**Published:** 1890-10-04

**Authors:** 


### Death Certificates and Murde .

The *Child's Guardian* for October 1st publishes an article by a police surgeon which shows painfully how cruelty and murder may be promoted by the heedless giving of Death Certificates on the part of medical men. For the instruction of non-professional readers we may be permitted to explain that when a person dies, who has been attended during his illness by a duly qualified practitioner, the medical man fills up and signs a formal certificate, stating that to the best of his belief and judgment the dead person died of a particular disease, which he names. Every medical man can be compelled by law to give such a certificate, unless he has reasonable grounds for believing that the deceased did not die from any cause which he can specify, or that death has been brought about by guilty means. In the large majority of cases the doctor is quite prepared to give a certificate of death from some natural cause or causes, because he has been in attendance on the patient for some time and has had every opportunity of forming a correct judgment. But there are many cases of sudden death where there has been no previous medical attendance. Even in some of these instances the doctor may feel justified in giving an ordinary certificate, as for example in the case of old people whom he may have known before to be suffering from heart disease, kidney affection, or the like.

There are, however, a considerable number of cases in which doctors give certificates that they are by no means justified in giving; and such certificates often have the effect of hiding away from dectection and punishment, cruelty and murder of the foulest kind. Here is one of several instances taken from the article in the *Child's Guardian* already referred to. It is the case of Reg. v. Flannigan and Higgins, and the details are as follows: On September 25th, 1883, Dr. Whitford, one of the district medical officers of the parish of Liverpool, was called to visit Thomas Higgins, a bricklayer's labourer, aged 45, whom he found in a cellar in one of the worst streets of Liverpool. The man had been drinking for two days, and had diarrhœa and felt "sickish." The pulse and temperature were normal. Medicine was given for the diarrhœa. Four days later the diarrhœa and sickness were worse, and were again treated. Three days afterwards the man's wife called at the surgery, stating that her husband was dead, and asked for a certificate. Dr. Whitford was surprised, as he had thought there was very little the matter with the man. The woman, however, said he had had "bloody" stools and died suddenly. The doctor thereupon certified "dysentery" as the cause of death. Next day Patrick Higgins, a brother of the deceased, called, and informed the doctor that his brother had been heavily insured by his widow and her sister, Catherine Flannigan. The doctor's suspicions were now thoroughly aroused, and he sent the brother to the coroner, and also saw that officer himself the following day. A post-mortem examination was made, and the viscera of the dead man were chemically examined. Not only had Thomas Higgins been insured for a hundred pounds and then poisoned, but John Flannigan, aged twenty-four, Margaret Jennings, eighteen, Mary Higgins, ten, and seven other victims, in all eleven persons, had been murdered in the same way by those two women. In each case insurance money had been the motive, and the sums insured had varied from a few pounds up to fifty and, as in Thomas Higgins' case, a hundred. In every one of these instances of deliberate poisoning there had been found a medical man to certify some natural cause of death. Had Dr. Whitford not been honest enough to carry the matter before the coroner even after he had given a certificate, those two women might have been still at their murderous work. They were, however, found guilty and hanged, without a single murmur of sympathy being raised on their behalf.

It is plain that if Liverpool may be taken as a fair sample of English towns generally, there are large numbers of deaths certified by medical men as being due to natural causes, which are really murders. We do not say that in every case the giving of such certificates can be avoided; but we do say that in many instances doctors are so careless, and so regardful of their own supposed interests, that they give certificates when they know full well that they ought not to do so. We do not know whether it occurs to them that in so doing they are conniving at murder; but that is certainly the case. It is a deep disgrace to an honourable profession that these things should be done; and we cannot but hope that Justice may by some means be able to lay her hands upon such guilty practitioners, and make them share the punishment of the vile creatures whose crimes they aid and abet. The public is under great obligations to the Society for the Prevention of Cruelty to Children for undertaking the painful duty of dragging such miserable facts to light. It is only by the discovery and prompt punishment of the guilty that others are deterred from continuing in careers of such shocking crime.

